# HABIT (Health visitors delivering Advice in Britain on Infant Toothbrushing): a qualitative exploration of the acceptability of a complex oral health intervention

**DOI:** 10.1186/s12875-022-01659-1

**Published:** 2022-03-26

**Authors:** Amrit Bhatti, Faye Wray, Ieva Eskytė, Kara A Gray-Burrows, Jenny Owen, Erin Giles, Timothy Zoltie, Victoria Smith, Sue Pavitt, Robert West, Rosemary RC McEachan, Zoe Marshman, Peter F Day

**Affiliations:** 1 University of Leeds, Leeds, United Kingdom England; 2 Bradford Institute for Health Research, Bradford, United Kingdom England; 3 University of Sheffield, Sheffield, United Kingdom England; 4 Bradford Community Dental Service, Bradford District Care NHS Foundation Trust, Bradford, United Kingdom England

**Keywords:** Dental caries, Behaviour change conversations, Oral health habits, Dental team, Health visitors, Intervention, Parents, Theory, Theoretical framework of acceptability, Acceptability, Qualitative, Framework analysis

## Abstract

**Background:**

To explore the acceptability of the oral health intervention, HABIT (Health visitors delivering Advice in Britain on Infant Toothbrushing) to parents with young children aged 9–12 months and health visitors.

**Methods:**

Following the delivery of the universal oral health intervention called HABIT, qualitative semi-structured interviews with parents and focus groups with health visitors were undertaken. Interviews were audio-recorded and transcribed. Health visitors completed self-reported diaries after delivering the HABIT intervention with parents. The qualitative data was analysed using framework analysis (guided by a theoretical framework of acceptability).

**Results:**

Seventeen parents were interviewed, and five health visitors and three nursery nurses participated in two focus groups. Parents reported health visitors to be ‘trusted’ and valued the reassurance provided during the HABIT visit. Health visitors found the HABIT training and resources useful and valued the consistency and increased confidence in undertaking oral health conversations. There were, however, challenges in changing behaviour where families faced competing demands on time and resources. Both health visitors and parents described the importance of the intervention's timing and suggested that multiple visits may be needed to support optimal oral health habits.

**Conclusion:**

The HABIT intervention was acceptable to parents and health visitors. Health visitors would welcome a further refinement to enhance intervention delivery that specifically achieves a balance between using a guided script and retaining the flexibility to adapt the conversation to suit the needs of individual families. This, in turn, will maximise impact and enable parents of young children to adopt and maintain optimal home-based oral health behaviours for their child.

**Supplementary Information:**

The online version contains supplementary material available at 10.1186/s12875-022-01659-1.

## Introduction

Globally, tooth decay is one of the most prevalent, yet preventable, diseases with approximately 621 million children having tooth decay experience within their primary teeth [1]. In England, 24% of children under the age of five experience tooth decay, with these figures increasing to 36% for children living in deprived parts of Yorkshire, a county in Northern England [2]. The impact of tooth decay is substantial, with the disease affecting children, their families and wider society [3–5].

Tooth decay, however, is preventable with regular toothbrushing with fluoride toothpaste and limiting sugary foods and drinks [6]. Within the UK, national guidance for children aged 0–3 years old includes parents or carers brushing the child’s teeth twice a day with a smear of fluoridated toothpaste (at least 1000 parts per million (ppm) fluoride) beginning from the eruption of the first tooth [1]. This is also known as parental supervised brushing (PSB). Throughout this paper, we will use the term “optimal oral health behaviours” to mean the adoption and maintenance of these oral health habits as outlined in UK guidelines. Furthermore, we will also use the term “oral health conversations” to capture the two-way discussion between health professionals and parents to support parents to establish optimal oral health behaviours.

Health visitors are key public health professionals with an important role in providing advice and promoting health to families of young children [7–9]. While the term “health visitor” is used within the UK, Denmark, Norway and Finland, the activities and models covered by health visiting can be found worldwide and have different names as outlined by the Institute of Health Visiting [10]. Every new mother in England receives a series of mandatory home visits from a member of the health visiting team (health visitor or trained nursery nurses), and this is usually the first point of contact with a professional within the health system [8, 11, 12]. When a child is aged 9-12 months, health visitors undertake a visit with the parents (either at home, nursery, clinic or health centre) to discuss child development, nutrition and obesity prevention, safety and oral health. In some local authority areas, parents also receive fluoride toothpaste and a toothbrush [13]. As part of this Healthy Child Programme [14], health visitors will ask whether the child is registered with a dentist and provide oral health advice. However, there is no mandatory oral health training and inconsistency in what and how any oral health advice is provided [15].

Health visitors will see parents before they visit the dentist and have regular contact with parents, which increases the opportunities to have oral health conversations at the earliest stages of a child’s life [8]. In some instances, mainly where access to dental clinics for new patients (e.g., young children) is limited or absent[16], health visitors may be the only source of oral health advice [15]. An exploration of health visitors experiences highlighted awareness of the risk factors for oral health and a willingness to provide advice [17]. However, Oge, Douglas [18] found that health visitors had limited specialised knowledge to help parents overcome barriers to toothbrushing and limiting sugary foods and drinks, and less than 25% were able to answer oral health questions appropriately. Similarly, Weston-Price et al. [12] and Eskyte et al. [15] identified a number of barriers, including gaps in knowledge and conflicting advice from other professionals. Mandatory universal health visits are a key opportunity for oral health conversations to be undertaken. The findings from existing studies, however, suggest effectiveness may be limited by a lack of specialised knowledge, training, and consistency of delivery for health visitors.

The current paper is part of a larger feasibility study, ‘Health Visitors delivering Advice in Britain on Infant Toothbrushing’ (HABIT). The HABIT intervention and feasibility study design are described within the methods section below, Supplementary materials (see Additional file 1) and a published protocol paper [11]. There is increasing recognition of the importance of qualitative methods in refining and optimising complex interventions [15]. Robust feasibility testing is critical for reducing research waste by ensuring that interventions are optimised before being tested in costly large-scale randomised controlled trials (RCTs) [15]. The current paper used qualitative methods to appraise the implementation of the HABIT intervention, focusing on the intervention’s acceptability to parents and health visitors. The feasibility study’s quantitative results are reported in a separate paper [19].

### Aim

To explore the HABIT intervention’s acceptability to 1) recipients - parents with young children aged 9–12 months and 2) those delivering the intervention - health visitors.

## Research design and methods

Throughout the paper, “health visitors” will be used as a collective term representing health visitors and nursery nurses who took part in the HABIT intervention.

### HABIT complex intervention design and a brief summary of the wider feasibility study

HABIT is embedded within the universal Healthy Child Programme visit undertaken by health visitors to all parents with a child aged 9-12 months. The HABIT intervention is a brief and structured oral health conversation between health visitors and parents. The feasibility study followed a mixed-method approach with four data collection periods (baseline, two-week follow-up, three-month follow-up and end of study interviews). Firstly, the health visitors were trained to deliver the HABIT intervention and use the supporting resources, including oral health training. A researcher visited parents following identification, recruitment and consenting to collect baseline data. The parent then received the HABIT intervention from the health visitor as part of the universal 9–12 month child development home visit.

Further data was collected from the home setting at two weeks and three months post-intervention; specifically, self-reported questionnaires assessing toothbrushing practices and their child’s diet, a dental examination; and video recorded observation of the parent brushing their child’s teeth. Following the delivery of the HABIT intervention, health visitors were asked to complete a brief diary describing the HABIT delivery and invited to take part in a focus group (See Additional file 2 for a copy of the diary). Three months after the HABIT intervention, the parents were invited to take part in a qualitative interview. A graphical summary of the HABIT procedure can be found below (Table 1).

**Table 1:** A graphical summary of the HABIT procedure

Timeline	Parents	Health visitors
HABIT training		a, b, c, d
Baseline	e	
HABIT intervention		f, g
Two-week follow up	h	
Three-month follow-up	h	
Focus groups/ interviews	i	j
a	HABIT training (full day) including: (1)Consent for health visitors to take part in the intervention and evaluation (2)Training on how to deliver the HABIT intervention and use the resources (3)How to use the dental models to explain toothbrushing skills (4)Informing the health visitors about the study design process and how the evaluation will take place (5)Explanation of how to fill in the diaries and any thoughts and refinements to the diaries (6)How to identify potential participants to take part in the intervention	
b	Health visitors identified parents who fit the inclusion criteria and asked if they were interested in participating in the study	
c	The health visitors team liaison sent potential participants the information sheet and consent form. Once consent had been received, participant information was released to the research team	
d	The research team contacted parents to arrange baseline data collection	
e	Data collection undertaken by the research team with parents: (1)Questionnaire, including parent and child demographics, self-reported toothbrushing habits, toothbrushing attitudes, dietary data and bedtime routines (2)Dental examination of the child – following British Association of the Study of Community Dentistry (BASCD) guidance, gingival inflammation and the Oral Hygiene Index (3)Parent child (dyad) toothbrushing interaction video-recorded	
f	HABIT intervention including: (1)Hand out a toothbrush and toothpaste (a standard practice for health visitors to give during the 9-12 month visit for children in this area) (2)Undertake toothbrushing demonstration (3)Explore parents’ oral health concerns (4)Showcase habit video, website and leaflet related to parent’s specific concern (5)Develop an action plan and write this on the leaflet	
g	Complete health visitor diary	
h	Follow up Data collection undertaken by the research team with parents: (1)Follow up Questionnaire, including parent and child demographics, self-reported toothbrushing habits, toothbrushing attitudes, dietary data and bedtime routines, attitudes about the intervention. (2)Dental examination of the child- British Association for the Study of Community Dentistry (BASCD) charting system, Gingival inflammation and Oral HYGIENE index (3)Parent child (dyad) toothbrushing interaction video-recorded	
i	Interviews: followed using interview guide A	
j	Focus groups: followed using interview guide B	

### Sample

All parents who received the HABIT intervention were invited face to face to take part in an interview after the final round of quantitative and clinical data collection, with 17 out of 28 agreeing to be interviewed (convenience sample). Reasons for non-participation included work commitments and language barriers. All participants were from the West Yorkshire region of England.

All health visitors who delivered the HABIT intervention were invited via email to take part in a focus group (convenience sample). Eight out of eleven agreed to take part, and in total, two focus groups were held. Reasons for non-participation were due to work commitments. Following the delivery of the HABIT intervention, 23 diaries were completed by health visitors and sent to a member of the research team (FW).

### Data collection

Multiple methods of data collection were employed to allow triangulation [20]. These included: (1) individual interviews with parents, (2) focus groups with health visitors, and (3) health visitor diaries.

All participants involved in the interviews/focus groups gave both verbal and written consent, and both followed a semi-structured interview guide (Additional file 3). The interview guides were constructed following recommendations by Ayala and Elder [21] and comprised of several topic areas: including thoughts on the intervention resources, training, and suggestions for improvement. The interviews were conducted by two researchers (AB & FW) with debriefing after each interview to provide opportunities to reflect upon the process and discuss any modifications to the topic guides. Both researchers also wrote notes after the interviews on matters of reflexivity and to aid interpretation of interviews at the analysis stage [22].

#### Individual interviews with parents

Individual interviews with parents (*n* =17) took place between October 2018 and February 2019. A member of the research team contacted the parents after the final round of data collection for the HABIT intervention to arrange a convenient time and date for the interview. The interviews took place within the home setting, and for most parents, their child was present. The interviews lasted between 20–45 minutes. Both researchers (AB & FW) were known to the parents as they took part in the previous quantitative and clinical data collection rounds.

#### Focus groups with health visitors

The focus groups took place in September 2018 and were carried out during the health visitors lunch breaks in a quiet private room in their place of work. Two focus groups were held, the first comprising of three health visitors and two nursery nurses, and the second comprising of one health visitor and two nursery nurses. In total, eight members of the health visiting team were interviewed, and both focus groups lasted for approximately one hour. The researcher (FW) was known to the health visitors as they had regular email contact with the health visitors throughout the study.

#### Health visitors’ diaries

After delivering each HABIT intervention, the health visitors were asked to complete a semi-structured diary (see Additional file 2). This recorded how the visit went, what oral health barriers were identified within the appointment, whether a toothbrushing demonstration was completed, and what resources were shown within the visit (website and leaflet). All data was anonymised. The completed health visitor diaries were sent to a member of the research team (FW). Out of 27 HABIT interventions, 23 diaries were completed.

### Analysis

All recordings of the interviews/focus groups were professionally transcribed. Data were anonymised and stored securely, along with field notes written at the end of the interviews. Transcripts from all interviews were checked by a member of the project team (FW). Data was analysed using Framework Analysis [23] using the following steps within an excel spreadsheet:

1. Familiarisation with the data
2. Coding the data using the theoretical framework of acceptability (TFA) by Sekhon, Cartwright & Francis [24]
3. Summarising data within the framework matrix
4. Interpreting the data/developing themes

After familiarisation (step 1), data was coded using the TFA (step 2). This framework captures the key seven acceptability constructs: affective attitude, burden, perceived effectiveness, ethicality, intervention coherence, opportunity costs, and self-efficacy (see Table 2 for more details). This allowed the researchers to explore data that focuses solely on the acceptability of the HABIT intervention.

**Table 2:** The theoretical framework of acceptability with definitions

Acceptability construct	Definition
Affective attitude	How participants felt about the HABIT intervention
Burden	The perceived amount of effort that is required to participate in the HABIT intervention
Ethicality	The extent to which the HABIT intervention has an optimal fit with the participants’ value system
Intervention coherence	The extent to which participants understand the intervention and how it works
Opportunity costs	The extent to which benefits, profits or values must be given up to engage in the HABIT intervention
Perceived effectiveness	The extent to which the HABIT intervention is perceived as likely to achieve its purpose
Self-efficacy	Participants’ confidence that they can perform the behaviours required to participate in the HABIT intervention.

Adapted from: Acceptability of healthcare interventions: an overview of reviews and development of a theoretical framework [25]

Qualitative summaries of the data were then developed across all seven constructs (step 3). This was to manage a large amount of data formed from the analysis, aligning with the nature of framework analysis [25]. This process facilitated the comparison of data within and across participants. The framework facilitated the development of higher-level themes with key characteristics of the data synthesised, and the dataset was interpreted as a whole (the development of themes). Theme development was undertaken by one researcher (AB), along with discussions with FW, to ensure consistency and coherency. There was flexibility in the analysis in which themes that did not fit with the framework were still included. Discussions between two research team members (FW & AB) ensured systematic coding of the data. This was an iterative, pragmatic approach in which themes were developed and changed over time and drew on both deductive and inductive processes. Both researchers (FW & AB) were females with a psychology background (PhD), experienced in conducting qualitative interviews. Both researchers were employed before the data collection stage.

The health visitor diaries supplemented the interviews and focus groups. The diaries were analysed at the end of data collection and after analysing the interviews and focus groups. One researcher (AB) read through the health visitors diaries and coded the data using the existing themes, which were developed from the individual interviews and focus groups [23]. At the end of the analysis process, multiple researchers (PD, AB, FW, KG-B and ZM) from different disciplines (paediatric dentistry, dental public health and psychology) were involved in peer debriefing and themes were then finalised. Transcripts were not returned to the participants, however, VS (a member of the health visiting team) reviewed the overall themes and findings to support member checking and enhance trustworthiness. The inclusion of the research team within this process enabled cross-validation and facilitated the exploration of issues that influenced the acceptability and feasibility of the intervention. All reached a consensus, aided investigator triangulation and ensured credibility and rigour until saturation had been reached.

## Results

### Overview

**Fig. 1:**
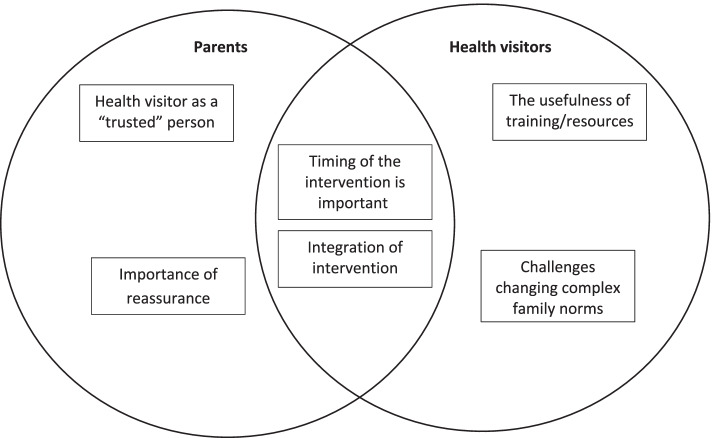
Themes from the parents and health visitors’ perspectives

Overall, health visitors and parents provided data to support the acceptability of the HABIT intervention for each of the seven constructs within the TFA. A summary table of the data within these acceptability constructs can be found in the supplementary materials (Additional file 4). When interpreting the dataset as a whole, two themes from parents and two themes from health visitors' perspectives were developed. Two cross-cutting themes were identified that were common to parents and health visitors. An overview of these themes can be found in Figure 1, and a tabulated summary of sub-themes can be found in Additional file 5.

### Key characteristics of the sample

#### Parents

The age of parents varied, with the average age of parents being 31. Many parents were born in the United Kingdom (*n* =21), with five parents born in Pakistan or Bangladesh and one parent from Poland. Parents were from the lower Index of Multiple Deprivation (IMD) centile groups, with 12 parents being in the most deprived decile, capturing the variety of backgrounds of individuals living in the area of West Yorkshire. All were female, and eleven of the participants were first-time mothers. Further demographic information can be found in our separate quantitative paper [19].

#### Health visitors

Health visitors had a wide range of experience. All were females and located across all postcodes in a city of West Yorkshire.

#### Parents’ perspectives

##### Health visitor as a trusted person

The theme of the ‘health visitor as a trusted person’ captured how parents felt about the health visitor being the facilitator of the intervention. Parents felt comfortable with the information being delivered by health visitors and thought they were the right person to deliver the intervention:

Obviously, you get a bond with them, don’t you, cause they’ve, like, you know, measured your baby and they’ve measured their everything, their head, this and that, and they come round from when they’ve been little for, like, now. So, you’re more comfortable, aren’t you, with them. - Parent 02

Multiple visits from the same health visitor helped build a strong rapport with the parent, especially if they visited the family to see older children. As such, discussing oral health in this context was particularly acceptable; parents were at ease and perceived this to be a ‘friendly chat’ with a familiar person. However, having a different health visitor at each visit was viewed as burdensome to parents. In this context, parents felt less at ease and had the burden of repeating information:

It’s just cause if you have previous concerns you’ve already spoken with the health visitor about and if you see her again she’s like “aw well has that improved?” cause she’s already seen you before. And she’s said you know she’ll recap on the last meeting and it feels more personalised and it’s a lot better cause then they know that child. And if there were concerns or things that stood out to them in one meeting they can sort of look at it in the following meeting and have a look whereas if it was someone different they would just look at it as if it was a new child and previous notes. - Parent 08

The narrative highlights how having a “personalised” visit is essential for parents and strengthens the notion of having a good rapport and establishing “bonds”. Having different health visitors caused frustration and could be a potential barrier to delivering oral health conversations.

##### Importance of reassurance

Parents who felt confident with their toothbrushing abilities were often experienced mothers with older children. Although these parents perceived additional information as unnecessary and non-essential, they stated how the intervention had provided reassurance that they were undertaking appropriate oral health behaviours.

They give you reassurance that he’s following the right stages cause maybe as a first-time parent, or a second time or even a third time just check.. is he doing okay, is this normal so kind of ask the first point of call is “is this normal?”- Parent 07

Equally, some parents with older children described how they had forgotten what to do and how to undertake the correct toothbrushing behaviours. As such, parents, especially first-time mothers, felt less confident with their abilities and needed reassurance. The parent within this narrative, for example, highlighted how she needed “confirmation” of when she should start toothbrushing:

…I suppose just the information about if there’s anything, in particular, you need to look out in terms of toothbrushes and toothpaste and things like that. Confirmation that you should just start when you should start brushing them. – Parent 19

Mothers, regardless of experience, stated how they were previously unsure of when to begin toothbrushing and for how long they should brush their child’s teeth. However, the intervention provided them with reassurance and advice on implementing the most appropriate oral health behaviours.

#### Health visitor perspectives

##### The usefulness of training/resources

Health visitors acknowledged the importance of delivering an effective oral health conversation. This health visitor, for example, highlighted how the HABIT training provided a consistent approach and a standardised way of supporting parents to adopt optimal oral health behaviours.

It’s been really useful to say, ‘well actually, yes, you know, I do know some of this but I actually, I have learnt quite a lot more on top of what I, I already knew about the, about the toothbrushing’… it’s important, it’s significant, but because we always tend to skirt around it, we’ve all been taught about different things, we’ve all attended different training sessions…. We don’t have a standard way of being taught about how to deliver oral health advice, although we do have training…. I’ve always found that to be a little bit wishy-washy. And that’s why maybe for me it’s been quite good that I’ve got something that I know is evidence-based. – Health visitor 01

In terms of the resources, positive feedback was provided by the health visitors and parents. Health visitors reported that parents responded well to visual demonstrations:

I thought the leaflet was quite good, and it was nice and compact and precise. There wasn’t a load of jargon on it. – Health visitor 02

Q: What did you like most about using the HABIT resources?

the leaflet with the reminders and prompts for parents. - Health visitor 05

Overall, the health visitors felt the resources were informative, simple, and visually pleasing. The little jargon meant that they thought the resources were suitable for a range of families. For some health visitors, the leaflets provided a useful prompt as it focused on several key topic areas. Other health visitors, however, stated they would like a guide for supporting conversations about oral health, such as a script or a crib sheet:

Sometimes you’ve got a lot of information to give. Without a script, things can be forgotten, or you can get side-tracked…- health visitor 01

What would you change in the content of the HABIT resources? Have a more structured info to give out. - Health visitors diary 04

Therefore, while some health visitors felt the leaflet alone was enough to support their conversations with parents, others would prefer further guidance on how to structure these conversations to ensure they have not missed key messages. Health visitors reported struggling to introduce the “action plan” on the leaflet and felt it could be perceived as patronising:

I think that was a bit, like, school teacher-ish. [action plan] - health visitor 04

Therefore, some health visitors were hesitant to use the action plan within their visit. This highlights that there were potential inconsistencies in how the intervention was delivered. Furthermore, implementation of the action plan could be perceived as a potential opportunity cost (i.e., to deliver the intervention, they had to use the action plan for every family, but did not believe it would be acceptable for all families):

If you get, like, a well-educated family, it’s like… it’s almost like you’re dictating that they’re gonna do it and, you know. And then the other end of the scale they think you’re telling them what to do. - Health visitor 08

##### Challenges changing complex family norms

Health visitors felt the intervention was suitable for most families. However, for some families, particularly those from disadvantaged backgrounds, health visitors felt establishing optimal oral health habits from an early age was more challenging. This was due to a variety of reasons including: cultural norms around diet and toothbrushing, and the perceived priority for families living in poverty (e.g., cost of healthy food, toothbrushes and toothpaste) and their wider concerns, such as housing, debt, unemployment and many other challenges. As such, health visitors questioned the perceived effectiveness of the intervention for these parents. There was the view that not many high-risk families, particularly those with complicated family dynamics; for instance, families known to social services; would engage with the intervention. This may lead to oral health being omitted within the visit owing to other health and social priorities:

Sometimes you go to a review, and they’ve got lots of issues... - Health visitor 07

Where there are other issues, such as safeguarding, having oral health discussions may not be the priority within the health visit or deemed appropriate. As such, there were challenges addressing complex family dynamics due to the perceived motivation of parents. One of the health visitors highlighted how she felt she had to “tread carefully” around the conversation of oral health, taking care not to be too directive:

So, you are going to agree to brush your child’s teeth twice a day, you are gonna agree to give them water or milk, and you are gonna give them a cup. It was just a bit, ‘cause you do have to tread really carefully with some of the families cause honestly, some of the families would tell you to [expletive] off. - Health visitor 06

Whilst changing these behaviours was viewed as difficult and sometimes met with resistance, the narratives show how health visitors used their knowledge and experience to adapt the intervention based upon their understanding and rapport with the family.

#### Cross-cutting themes

Two themes were developed, which represented experiences shared by parents and health visitors. These are presented as cross-cutting themes.

##### Timing of the intervention is important

Health visitors and parents stated the importance of the intervention being delivered at the appropriate time when the child’s teeth were starting to erupt:

You know, their cheeks are getting red. And you know that you can’t see anything, their teeth haven’t erupted yet, but they’re not too far away. - Health visitor 09

This was identified to be the optimal time to deliver oral health conversations for parents and health visitors. For some parents, their children’s teeth had already erupted before the HABIT intervention. This meant that parents had already started undertaking toothbrushing behaviours and some suggested that the information was less valuable at this stage. The parent below discusses how they would have liked to have the knowledge of how to perform toothbrushing correctly before their child’s first tooth had erupted:

…when she come out, he had his first tooth. So, he were a bit earlier with his teething, but, see he got his first tooth before six months, and I think the four, five, six-month mark would probably be better [for a visit]. If I had earlier, I wouldn’t have done what I were doing in the first place. - Parent 04

Health visitors suggested discussing oral health within the three to four-month visit so that parents were aware before the teeth erupted. This could be followed up in more depth at their next visit:

It’d be good to do it, I think to bring it up at the three to four-month. But also, I think we do need to mention it at the nine to twelve months and then again…- Health visitor 06

Health visitors highlighted the need to continuously reinforce oral health throughout each interaction to ensure optimal oral health habits have been established and maintained. This was especially needed when the child became older and potentially more resistant to toothbrushing routines:

I think it might be useful to give tips of how he might actually, he might move around or he might do this and try, like little tips of different sort of things that we could do in scenarios basically. Cause he wouldn’t really, he didn’t like it at the beginning and he would just bite and after a few months, it weren’t working he would just bite on the brush and now he kind of like, he’ll walk around and play with the toothbrush and just put it in his mouth and bite on it and just do what he wants with it. – Parent 17

Some parents found it challenging to implement parental supervised brushing (PSB) at this particular age (over 1-year-old) because they struggled to establish how far they could “push” their child while also keeping toothbrushing a positive experience. This, in turn, could disrupt the flow of existing routines and, therefore, was viewed as burdensome. Guidance and reassurance in this area might have been useful in which health visitors can offer advice on how to keep toothbrushing a positive experience while also highlighting the appropriate PSB skills when their child is resistant. This parent, for example, wanted to ensure she had done enough to clean her child’s teeth without causing too much distress:

Maybe a bit more, well what’s the minimum I need to do? Literally get some fluoride on the teeth. - Parent 16

Often, the resistance of the child when toothbrushing, impacted the parent’s confidence and left them feeling guilty when they could not undertake what they perceived to be a “good brush”. Therefore, providing parents with basic advice of the “minimum amount” was viewed as important.

##### Integration of the intervention

Health visitors reported that the intervention complimented their existing conversations regarding oral health and dietary behaviours:

I think we deliver it anyway. We probably just went into it in more depth. - Health visitor 01

Furthermore, some health visitors felt that the intervention complimented the length of their visit and allowed them to focus on one key oral health message that applied to parents:

The fact that you can go on that, you can dip into any of them. If you’re interested in one section then you can go, you can watch that. And, you know, I, I found they was quite useful. I liked the fact that they weren’t very long, you know. I think the longest one was the one about behaviour, the video. But, but I think that was useful in that [laughs] quite a few mums would talk about behaviour and children…- health visitor 08

However, for others, the HABIT intervention went beyond the anticipated five minutes:

Interviewer: How long was it taking you to deliver the habit side of things in your visits would you say?

Health visitor 05: A good hour.

This was reflected in the health visitor diaries, where one health visitor noted the oral health discussion to last up to 50 minutes and demonstrated that all aspects of oral health were discussed, rather than focusing on one key topic identified by the parent. This may suggest health visitors felt obliged to go through all areas regarding oral health such as diet, managing behaviours and toothbrushing:

Question: Which further tooth brushing skills did you discuss?

Using 1000ppm fluoride toothpaste, cleaning together, cleaning all surfaces especially back teeth twice a day, night cleaning important , supporting cleaning until 7 years, be aware of sugar recovery time, sharing information with family member who care for the children...- Health visitors diary 06

All aspects of the HABIT information guide were discussed- Health visitors diary 07

While the intervention fits well within the health visitor’s standard delivery, how well it fits into family life from the parent’s perspectives varied. Some parents found implementing PSB to be challenging:

…every evening we definitely do it. Most mornings I try do it after breakfast. Sometimes, if I’m in a massive rush to get to work and he’s going to nursery we don’t have time. But more often than not we do. Sometimes it’s a have breakfast, fly out the door situation you see…- Parent 12

Getting into the habit of toothbrushing might disrupt the flow of existing routines, particularly if the child became upset or was resistant to toothbrushing. Parents generally had very good intentions and saw the value of PSB. It was, on the other hand, sometimes deprioritised in the context of a busy family life with competing demands, such as getting older siblings to school on time.

Having older children with an established routine with regards to oral health was a facilitator to parents taking on board messages from the HABIT intervention:

“I think with having [name of older child], I don’t know, I think for me, I just, I dunno, you know to brush their teeth. So as soon as both of them got teeth, it was just an obvious thing.”-Parent 13

The HABIT leaflet also provided parents with the opportunities to pass on information to the wider family:

But they concentrated a lot more on the toothbrushing, which was really good. So it was good for me cause I know how important it is but you don’t know how to explain to other people. Sometime like my mother-in-law or someone [unclear words 0:05:03]. So, it was really good the way they explained it so I could tell my mother-in-law, ‘look’. And I gave her that little booklet as well and I said, ‘look this will help’. So, it was really good. – Parent 09

While the intervention appeared to fit in well for families and health visitors, there were also broader barriers that challenged how health visitors undertook their oral health conversations. This included parents being able to access dental practices, despite the resources highlighting the need for a dental check by one year old [16, 26]:

That’s the, you think [name of city] ’s, it’s so deprived isn’t it, and we’ve got far less dentists…. It’s okay if people either are willing to travel but also can afford to travel - Health visitor 07

As such, health visitors stated how they felt as though oral health conversations should not just fall to their team and that there were multiple approaches needed to address oral health:

I think, well it seems it’s a multi-professional thing. I think we shouldn’t be the ones who should be expected to be, be doing this, not just on our own. I mean gosh, you know, the nurseries, there are nurseries out there. You know, okay we’re doing this at nine, nine to twelve months old but some children go to day nurseries, and their parents go to work, childminders. - Health visitor 06

Health visitors suggested the need for a combined effort from other organisations, such as nurseries, to support parents and continuously reinforce oral health conversations and optimal oral health behaviours throughout each interaction. This health visitor also suggested that there are additional complexities, such as parents going to work and leaving their child with childminders. Hence, other collaborative efforts may be needed.

## Discussion

The current study explored the acceptability of the HABIT intervention for parents with young children aged 9–12 months old and health visitors. Overall, the findings show the HABIT intervention was acceptable to parents and health visitors, but several contextual factors influenced the level of acceptability. These included establishing a relationship with the health visitors, the timing of the visit, family dynamics, and the need for consistency and availability of support from other professionals. In agreement with earlier research [27], the role of the health visitors in providing oral health messages, and undertaking oral health conversations, was perceived to be important by parents and health visitors. This reinforces the important role health visitors have in supporting optimal oral health behaviours and limiting tooth decay in young children [28]. The health visitors appeared to value the importance and utility of training to support oral health conversations, especially given the lack of appropriate oral health training, as identified by previous research [8, 17, 29, 30].

The Healthy Child Programme [31], delivered by health visitors, follows a Universal Proportionalism model and includes universal, targeted pathways and specialist services [32]. HABIT is designed to be delivered as part of the universal pathway to all parents with children aged 9-12 months. With only a few teeth erupted at this age and no decay evident [19], establishing optimal oral health routines is critical for developing lifelong habits and preventing tooth decay. This was demonstrated in the “Importance of reassurance” theme with parents welcoming the HABIT conversation and benefitting from conversations around optimal oral health behaviours. The diverse sample recruited to the study included families whose child was at high risk of future decay. Indeed, earlier research [33] has shown the accuracy of health visitors subjective “gut” feeling at identifying one year old children who were likely to develop tooth decay by the age of four. This was identified in the theme “Challenges changing complex family norms” and difficulties health visitors reported in supporting families with multiple urgent priorities and the limited value some families place on good oral health. These families may reach the threshold for targeted pathways within the Healthy Child Programme. These enhanced support pathways provide opportunities for further oral health conversations, such as those embedded within evidence-based structured prevention-focused programmes (e.g., Maternal Early Childhood Sustained Home Visiting [34]). Within the confines of a universal home visit, a “one-off” HABIT oral health conversation was challenging when delivered to families with complex dynamics and multiple pressing priorities. Importantly, this finding reinforces the need to improve the HABIT training for health visitors when confronted with resistance. For example, the need to move away from the delivery of one-way oral health education towards oral health conversations utilising strategies taken from motivational interviewing (e.g., rolling with resistance) methods. Despite these challenges, the parallel quantitative evaluation of the HABIT interventions [19] found a strong trend of improved oral health behaviours.

While dental teams can support parents to adopt optimal oral health behaviours for children, there are significant barriers and inequalities in dental access [16]. Despite national campaigns for children to attend the dentist before their first birthday [26] and health visitors promoting attendance, within our study location, less than 5% of children attend the dentist by the age of one [35]. Consequently, as highlighted in the theme “Integration of the intervention”, some health visitors felt frustrated by the lack of access for young children and felt a significant responsibility for supporting optimal oral health behaviour. Local initiatives are needed to facilitate ‘joined-up care’ and communication between health visitors and dental practices. This includes the co-ordination of consistent oral health conversations across early year sectors and the importance of dental teams embracing their wider role in the community beyond their dental practice to work with health visitors [36]. Both upstream and downstream approaches can facilitate access and communication [37].

### Implications for intervention development

Firstly, in refining the intervention, it is necessary to consider the flexibility of the approach. On the one hand, health visitors reported that they liked that the HABIT intervention brought uniformity and consistency to delivery. Indeed, some suggested that they would have liked an even more structured ‘script’ to guide the oral health conversation. On the other hand, some suggested the impact of the intervention may not be universal, notably that for families with complex needs, where further support and additional visits may be needed in conjunction with further health visitor training around supporting “resistant” families.

There is a balance between uniformity and flexibility within the training and delivery of the intervention. For example, while there is a good evidence-base for the effectiveness of action plans [38], this may fit with the health visitors’ standardised format or their style of delivery, and some reported not using the action plan. Therefore, further training is required to make more transparent the purpose of the “fixed” components of the intervention (such as the action plans and toothbrushing demonstrations) and “flexible” components of the intervention, which are tailored to the parents’ needs and the context within which the family operates.

It was anticipated that the HABIT conversation would last approximately ten minutes out of a one-hour standard health visit. For some health visitors, the conversations took longer than expected to deliver. The diaries suggest that health visitors felt obliged to go through all areas regarding oral health, such as diet, managing behaviours and toothbrushing. This is similar to previous studies in that health visitors felt as though they needed to provide additional detailed oral health information [29], rather than reinforcing one key oral health message [17]. The findings show variance in how the intervention was delivered, and there should be an emphasis on avoiding information overload on parents. Furthermore, further training and support is needed for health visitors in flexible and tailored intervention delivery, so they feel delivering the intervention does not take too much of their time and therefore is not viewed as burdensome.

Finally, the findings also identify that a ‘drip’ approach may be needed to maximise the impact of these oral health conversations and align them with the needs of the parent at their child’s particular stage of development. Parents’ narratives suggested that some children became more resistant to toothbrushing between 12-15 months old. Their reflection was that at the time of the HABIT intervention, PSB was relatively easy to perform, but over the following three months, the child’s wish for independence and resistance to PSB became more apparent. Therefore, HABIT resources could include anticipatory guidance to parents, which is then followed by periodic reminders (such as text messages or telephone calls) in conjunction with online resources. This, in turn, may help reinforce messages at a more appropriate time which matches their child’s developmental needs.

### Strengths and limitations

At present, there is a dearth of studies that have followed a complex intervention methodology to develop and evaluate oral health interventions delivered by health visitors [39]. The strengths of the study include the use of a robust and theoretically underpinned framework (i.e., TFA) to explore intervention acceptability. As highlighted, the data was coded across all seven constructs of acceptability. A key stage of the framework approach is going beyond the data and developing themes by comparing data within and across participants [23]. By doing so, the views of each participant remain connected to other aspects of their account so that the context of the individual’s views is not lost [40]. In turn, these themes capture complex layers of meaning and understanding, which went beyond exploring how the data fits within acceptability constructs of the TFA.

The current study has three main limitations. Firstly, the views presented may differ to parents who dropped out of the study or declined to be interviewed. Similarly, health visitors who did not attend the focus groups may differ from those included in this paper. Secondly, for the intervention and data collection, families were required to speak English. Although for non-English speaking families, an interpreter may be present within their universal health visit, the current study did not have the funding to hire interpreters. As such, these findings are not generalisable to non-English-speaking families and highlight the need for further work to widen the access of the habit resources, which is currently being undertaken. Thirdly, although the resources were co-designed in plain English, the HABIT materials were only accessible to parents who did not have sensory (vision and hearing in particular) or cognitive impairments. Parents may need the information to be provided in alternative formats such as Braille, large print, audio, or easy read. Respectively, in addition to making the HABIT intervention accessible to non-English speaking families, the resources should be provided in various alternative formats that are universally accessible. For pragmatic reasons, this widening of accessibility is intended as phase two of the project, once feasibility is established. Further funding (https://www.betterstartbradford.org.uk/oral-health/university-of-leeds-to-work-with-better-start-bradford-to-improve-oral-health-in-0-3s/) has been secured to address both the parent-facing limitations as well as refine the HABIT training and support provided to health visitors delivering the intervention.

## Conclusion

This qualitative exploration allowed for a more nuanced understanding of the intervention's acceptability, which has direct implications for the optimisation of the intervention. Further iterations include the capacity for the intervention to be delivered at different time points and the need to retain the flexibility to adapt the messages to suit the needs of the individual families. Such adaptations will be addressed following further funding to refine the resources and training for health visitors in preparation for a definitive study.

## Supplementary Information

**Additional file 1.** Intervention development using TIDieR (template for intervention description and replication) checklist.

**Additional file 2.** Self-reported health visitor diaries.

**Additional file 3.** Topic guides (parents and health visitors).

**Additional file 4.** A summary of the results from the HABIT intervention reported using constructs from the Theoretical Framework of Acceptability (TFA) by Sekhon.

**Additional file 5.** A tabulated summary of themes and subthemes.

## Data Availability

The datasets used and analysed during the current study are available from the corresponding author on reasonable request.

## Abbreviations

HABIT: Health Visitors delivering Advice in Britain on Infant Toothbrushing
NIHR: National Institute for Health Research
NIHR ARC YH: NIHR Applied Research Collaborations Yorkshire and Humber
RCT: randomised controlled trials
TFA: theoretical framework of acceptability
PSB: parental supervised brushing
