# Supplementary material for: HABIT (Health visitors delivering Advice in Britain on Infant Toothbrushing): a qualitative exploration of the acceptability of a complex oral health intervention
Source: BMC Prim Care. 2022 Mar 26;23:55. doi: 10.1186/s12875-022-01659-1 (PMC8962587; doi:10.1186/s12875-022-01659-1)
Supplement: Supplementary file 1 — Additional file 1. Intervention development using TIDieR (template for intervention description and replication) checklist. [file 12875_2022_1659_MOESM1_ESM.docx]

Additional File 1: Intervention development using TIDieR (template for intervention description and replication) checklist (36).

Please note, the term, “health visitors” will be used collectively to represent health visitors and nursery nurses who took part in the HABIT intervention.

| Brief name of intervention | HABIT (Health visitors delivering Advice in Britain on Infant Toothbrushing) |
| --- | --- |
| Why (rationale, theory, goal) | The impact of tooth decay is significant, with the disease affecting the child, their family and wider society. Health visitors and their teams are key public health professionals with an important role in providing advice and promoting health behaviours to families of young children. However, existing research has found wide variation in practice. Some health visitors have inconsistent access to training and limited practical knowledge on how to support parents overcome barriers to toothbrushing and limiting sugary foods and drinks.  HABIT is a co-designed complex intervention to support health visitors’ oral health conversations with parents during the 9-12 month universal developmental home visit. The HABIT intervention delivered by health visitors, aims to empower parents to establish optimal oral health behaviours.  Following a complex intervention methodology, our multi-disciplinary team co-designed digital and paper-based training and resources with health visitors and parents of children aged 9–24 months. The intervention comprised of two components: (A) training for health visitors to deliver the HABIT intervention and (B) HABIT resources for parents, including a website, videos, toothbrushing demonstration and a paper-based leaflet with an action plan.  Details describing how the HABIT intervention was co-designed are described in Owen et al. (in preparation). |
| What  1. Materials for intervention and training (access to materials)  2. Procedures (describe activities and support activities) | 1. All health visitors delivering the intervention attended a one-day training course. This included updates and guided discussions around oral health knowledge through viewing novel television-based programmes designed to support early-years professionals oral health knowledge ([www.soap.media](http://www.soap.media)). The HABIT resource ‘pack’ was issued; including the standard delivery protocol (outlined below), HABIT leaflets, toothbrushing models, HABIT website link ([www.toothbrushinghabit.com](http://www.toothbrushinghabit.com)) and self-reported diaries. These different resources were reviewed and discussed with health visitors in conjunction with how to use them as part of an effective oral health conversation using tools from motivational interviewing. Behaviour change techniques were incorporated throughout the digital and paper-based HABIT resources.  2. At the first training event, health visitors, research team members and dental professionals agreed on a standard delivery protocol. This included: (1) Identifying parental concern about oral health and assessing the parent’s motivation. (2) Engaging in an oral health conversation tailored to the parents needs and concerns using the HABIT leaflet as a guide. (3) Showing the HABIT website and appropriate video matched to parents’ concern. (4) A toothbrushing demonstration (5) Developing a tailored action plan with the parent (5) Giving the parent the HABIT leaflet with a written action plan, toothbrush and toothpaste. (6) Completing health visitor diary. |
| Who provided (describe expertise, background, specific training) | The intervention is designed for delivery by health visiting teams (including health visitors and nursery nurses) following additional HABIT training. |
| How (modes of delivery, e.g., face to face/individual group) | HABIT is delivered as part of the Healthy Child Programme at the 9-12 month universal development review. The Healthy Child Programme is a universal intervention delivered by health visitors to all parents with young children aged 0-24 months. There are a minimum of five home visits of which, one is at the 9-12 month timepoint. |
| Where (types of locations) | Parental homes/clinics/children’s centres |
| When and how much (how often is the intervention delivered, duration) | For purposes of the feasibility study, the HABIT intervention was delivered once. |
| Tailoring (how will the intervention be individualised) | The conversation is guided by parents, who self-identify barriers to oral health and solutions. Individualised action-plan is created. |
| Modifications (any changes during the study) | Reported throughout the manuscript. |
| How well  1. Intervention fidelity assessed by    2. Actual adherence | The results of the feasibility study are reported in two papers (Bhatti et al. 2021 and Giles et al. 2021). These papers described the acceptability of the HABIT intervention to parents and health visitors, the feasibility of delivery, and the intervention's impact on oral health behaviours of young children. A further paper (Owen et al. 2021) describes in detail the co-design of the HABIT intervention.  1. Fidelity was assessed through qualitative interviews and focus groups with parents and health visitors in conjunction with reviewing the diaries completed by health visitors following intervention delivery.  2. Actual adherence is reported in the qualitative paper reported in Bhatti et al. 2021. |
