# Supplementary material for: HABIT (Health visitors delivering Advice in Britain on Infant Toothbrushing): a qualitative exploration of the acceptability of a complex oral health intervention
Source: BMC Prim Care. 2022 Mar 26;23:55. doi: 10.1186/s12875-022-01659-1 (PMC8962587; doi:10.1186/s12875-022-01659-1)
Supplement: Supplementary file 2 — Additional file 2. Self-reported health visitor diaries. [file 12875_2022_1659_MOESM2_ESM.docx]

Additional file 2: Self-reported health visitor diaries

Outline of the health visitor diary

HABIT: A study to explore and improve oral health and tooth brushing advice given by Health Visitors

Information about parent/child to whom the HABIT oral health advice has been provided:

Date of the visit: ………………………………………………………………………………………………………………………..………….…..

Who did you deliver the information to (e.g. mum, dad, both?) ……………………………………………………………….

How many times have you delivered the HABIT oral health advice? (family number 1,2,3,4,5)? …………….

…………………………………………………………………………………………………………………………………………………………………..

Generic information about the home visit:

What were the general health topics most important to the parent? ………………………………………………………….

……………………………………………………………………………………………………………………………………………………………………………..…………………………………………………………………………………………………………………………………………….…………………

How long did the home visit take? ………………………………………………………….…………………………………………………….

How long did the discussion about baby’s oral health take? ………………………………………………………………………...

The HABIT intervention has four components, please record your experiences of delivering the intervention and the parent’s receptivity to the different components.

PART 1 – HANDING OUT THE DENTAL PACK AND INITIATING AN ORAL HEALTH CONVERSATION

A. Did you ask if the parent has started brushing their baby’s teeth? Yes/ No

If No, continue with Part 2

If Yes, provide more details on the following aspects:

• Does the parent brush baby’s teeth? Yes/ No

• If yes, how often do they brush baby’s teeth? ………………………………………………………………..………………...

• Who else in the family brushes baby’s teeth? …………………………………………………………………………………...

• What oral health products does the family use for their baby (e.g. type of toothpaste, a baby sized toothbrush)? ……………………………………………………………………………………………………….…………………………….

…………………………………………………………………………………………………………………………………………………………..

• Has the family ever taken their baby for a dental check-up? Yes/ No

PART 2 – TOOTHBRUSHING DEMONSTRATION

B. Did you ask parents to brush their baby’s teeth? Yes/ No

If No, continue with Part 3

If Yes, provide more details on the following aspects:

• Did the parent demonstrate basic toothbrushing knowledge (used right size toothbrush, right amount and strength of toothpaste, brushed all surfaces)? Provide more details ……………………………..

……………………………………………………………………………………………………………………………………………………….....

…………………………………………………………………………………………………………………………………………………………...

• Did you demonstrate toothbrushing technique with the baby? Yes/ No

Or did you use the plastic “clacker teeth and toothbrush” Yes/No

• How did the parent react to being asked to brush and/or the demonstration? ………………………………..

.................................................................................................................................................................

…………………………………………………………………………………………………………………………………………………………...

PART 3 - DISCUSSION ON FURTHER TOOTHBRUSHING AND/OR DIET AND SIGNPOSTING TO HABIT LEAFLET AND WEBSITE

C. Did you discuss further toothbrushing skills and/or diet? Yes/ No

If No, continue with Question D

• What were the most important issues to parents regarding baby oral health? …………………………………

…………………………………………………………………………………………………………………………………………………………..

…………………………………………………………………………………………………………………………………………………………..

If Yes, please provide more details on the following aspects:

• Which further toothbrushing skills did you discuss? ………………………………………………….........................

…………………………………………………………………………………………………………………………………………………………..

• Which aspects of diet did you discuss? ……………………………………………………………………………………………..

…………………………………………………………………………………………………………………………………………………………..

• How was your advice received? …………………………………………………………………………………………………………

…………………………………………………………………………………………………………………………………………………………...

D. Did you show parents the HABIT leaflet and use it to reinforce key messages? Yes/ No

Did you signpost parents to the HABIT website? Yes/ No

Did you take parents onto the HABIT website on their phone/tablet/computer or yours? Yes/ No

If No to all three questions, please continue with PART 4

If Yes, please identify which areas of the HABIT resources you concentrated on:

• No second chance (Motivation)

• Toothbrushing know-how (knowledge)

• Toothbrushing tips (skills)

• Managing behaviour

• Managing the wider social environment

• Diet know-how (knowledge)

• Which are was the most important for the parent when talking about these barriers? …………………………………………………………………………………………………………………………………………………………….…………………………………………………………………………………………………………………………………………

• What was the parent’s overall reaction to the HABIT resources? ……………………………………………………

………………………………………………………………….……………………………………………………………………………………..

…………………………………………………………………………………………………………………………………………………………

• Was the parent able to understand HABIT resources? Yes/ No and which components did they engage with most? …………………………………………………………………………………………………………………………….

…………………………………………………………………………………………………………………………………………………………...

• Did the parent disagree with any part of the HABIT resources? ………………………..……………………………….

…………………………………………………………………………………………………………………………………………………………...

• Do you feel a further visit is needed to support parents to adopt good oral health behaviours?

Yes/ No

PART 4 – ACTION PLAN

Did you complete an action plan with the parents? Yes/ No

If No, please go to Part 5

If Yes, which of the action plans were agreed upon in the Action Plan?

Brush my baby’s teeth twice a day with fluoride toothpaste

Use a smear of fluoride toothpaste

Try to make brushing fun

Try not to give up if the baby refuses to have their teeth brushed

Won’t give sweetened foods or drinks an hour before bedtime

Try to stick to three meals and two healthy snacks a day

Try to stick to milk and water to drink

Try to move onto a free-flow cup

How does the parent intend to do this? ………………………………………………………………………………………………………..

……………………………………………………………………………………………………………………………………………………………………….

PART 5 – Health Visitor feedback on delivering the oral health conversation and using the HABIT resources

• Did you feel comfortable delivering the oral health conversation and using the resources?.................

…………………………………………………………………………………………………………………………………………………………...

• What did you like most about using the HABIT resources? ………………………………………………………………...

…………………………………………………………………………………………………………………………………………………………...

…………………………………………………………………………………………………………………………………………………………...

• What did you like least about using the HABIT resources? ………………………………………………………………..

…………………………………………………………………………………………………………………………………………………………..

• What would you change in the content of the HABIT resources? ……………………………………………………

…………………………………………………………………………………………………………………………………………………………………………………………………………………………………………………………………………………………………………………..

• Did you have sufficient time for the oral health conversation, including signposting the HABIT resources? Yes/ No

• If No, how much time would you need for the oral health conversation? …………………………...............

Do you have any other comments about the HABIT oral health advice?

…………………………………………………………………………………………………………………………………………………………...............

…………………………………………………………………………………………………………………………………………………………...............

…………………………………………………………………………………………………………………………………………………………...............

…………………………………………………………………………………………………………………………………………………………...............

…………………………………………………………………………………………………………………………………………………………...............

…………………………………………………………………………………………………………………………………………………………...............

…………………………………………………………………………………………………………………………………………………………...............

…………………………………………………………………………………………………………………………………………………………...............

…………………………………………………………………………………………………………………………………………………………...............

…………………………………………………………………………………………………………………………………………………………...............

…………………………………………………………………………………………………………………………………………………………...............

…………………………………………………………………………………………………………………………………………………………...............

…………………………………………………………………………………………………………………………………………………………...............

…………………………………………………………………………………………………………………………………………………………...............

…………………………………………………………………………………………………………………………………………………………...............

………………………………………………………………………………………………………………………………………………………….............

Thank you for completing the diary for this family.
