# Supplementary material for: HABIT (Health visitors delivering Advice in Britain on Infant Toothbrushing): a qualitative exploration of the acceptability of a complex oral health intervention
Source: BMC Prim Care. 2022 Mar 26;23:55. doi: 10.1186/s12875-022-01659-1 (PMC8962587; doi:10.1186/s12875-022-01659-1)
Supplement: Supplementary file 3 — Additional file 3. Topic guides (parents and health visitors). [file 12875_2022_1659_MOESM3_ESM.docx]

Additional File 3: Topic guides (parents and health visitors)

**Parent interview guide**

FUN ACTIVITY

1. Let’s start with a fun activity. I am going to ask each of you to fill in this sentence.

• When I want to learn how to do something, I ______________________________________.

• Three words to describe the person who has helped me most in life are _________________.

INTRODUCTORY QUESTIONS

2. What comes to mind when you think of making changes to your baby’s toothbrushing habits?

• PROBE: Your own toothbrushing habits?

3. How can we help you make changes in your baby’s toothbrushing habits?

• PROBE: What type of information would be most helpful to receive?

TRANSITION QUESTIONS

4. Thinking about your own family, if we assigned someone to work with you – like a Health Visitor or a promotor – describe what type of person would be most helpful?

• PROBE: What specific things could s/he do that would be most helpful?

• PROBE: How old should the person be?

• PROBE: Should the person be male or female?

• PROBE: Should the person have kids? Be married?

• PROBE: Should the person be Latino/Hispanic/Mexican/Indian/Polish?

DEPTH QUESTIONS

Recently you received a universal home visit the Health Visitor.

• What topics did you discuss?

• What was the most important for you as a parent?

• Who has initiated the discussions: you or the Health Visitor?

• Did you find the discussions useful?

One of the discussed topics was your baby’s oral health.

• How long did the discussion take?

• What barriers to oral health did you discuss?

• Were the resources helpful for better understanding baby’s oral health needs?

• What was good? Why?

• What needs to be improved? Why?

• What could we loose? Why?

• Were the resources easy to understand?

• Did you feel comfortable and confident when seeing and using the resources?

• Do the resources address your needs and preferences regarding baby’s oral health?

Before delivering oral health advice to you, the Health Visitor received training on how to use HABIT resources.

• Did the Health Visitor know the resources well?

• Was the Health Visitor confident and comfortable when using the resources?

Do you follow the advice provided by the Health Visitor?

• Do you feel encouraged or discouraged to take care after your child’s teeth?

• Do you feel confident following the advice provided by the Health Visitor?

CLOSING QUESTION

We have asked a lot of question of you. Now we want to turn the tables a bit. What questions do you have of us that are related to the provision of oral health advice?

Thank you to everyone who made an effort to be here tonight.

**Health visitor topic guide**

FUN ACTIVITY

1. Let’s start with a fun activity. I am going to ask each of you to fill in this sentence.

• When I want to advise parents of something, I __________________________________________.

• Three words to describe the family that you have helped me most in your career as a Health Visitor are ______________________________________________________________________________.

INTRODUCTORY QUESTIONS

2. What comes to your mind when you think of making changes in parent’s infant toothbrushing habits?

• PROBE: Your own toothbrushing habits?

3. How can we help you to make changes in parent’s infant toothbrushing habits?

• PROBE: What type of information would be the most helpful?

• PROBE: What type of resources would be the most helpful?

TRANSITION QUESTIONS

4. Think about your own professional practice: if you are assigned a family of infant aged 9-12 months to provide an oral health advice for – describe what type of parent would be most helpful?

• PROBE: What specific things could s/he do that would be most helpful?

• PROBE: How old should the person be?

• PROBE: Should the person be male or female?

• PROBE: How many kids should the person have? Be married?

• PROBE: Should the person be Latino/Hispanic/Mexican/Indian/Polish/etc.?

DEPTH QUESTIONS

During the home visit at 9-12 months of an infant you as a Health Visitors deliver an oral health advice.

5. What is the normal practice of providing oral health advice to parents of infants aged 9-12 months?

• How long does it usually take?

• What topics do you usually discuss?

• Who usually initiates the discussion, you or a parent?

• Do parents find the discussions useful?

6. What was the practice of using HABIT resources and providing oral health advice?

• How long did the intervention take?

• What barriers to oral health did you discuss?

• What was your experience of using HABIT resources when discussing these barriers? Were they helpful?

• What was good? Why?

• What needs to be improved? Why?

• What could we loose? Why?

• Did you feel comfortable and confident using the HABIT resources?

• Do the HABIT resources and oral health intervention ‘fit’ into the mandatory home visit?

• Are the resources feasible? Do they meet the actual needs of parents?

• How did the parents react to the HABIT resources? Which resource did they find to be most useful?

7. What is your opinion about the training on how to use HABIT resources?

• Was it helpful? Which parts of the training did you enjoy most? Why?

• Did the training contribute to expanding your knowledge about oral health? How?

• Was the training useful for using HABIT resources in the home visits? How?

• What should be improved? Why?

• Are there any other aspects that should be included in the training?

• What could we loose?

CLOSING QUESTION

We have asked a lot of question of you. Now we want to turn the tables a bit. What questions do you have of us that are related to the provision of oral health advice?

Thank you to everyone who made an effort to be here tonight.
