# Supplementary material for: HABIT (Health visitors delivering Advice in Britain on Infant Toothbrushing): a qualitative exploration of the acceptability of a complex oral health intervention
Source: BMC Prim Care. 2022 Mar 26;23:55. doi: 10.1186/s12875-022-01659-1 (PMC8962587; doi:10.1186/s12875-022-01659-1)
Supplement: Supplementary file 4 — Additional file 4. A summary of the results from the HABIT intervention reported using constructs from the Theoretical Framework of Acceptability (TFA) by Sekhon. [file 12875_2022_1659_MOESM4_ESM.docx]

Additional file 4: A summary of the results from the HABIT intervention reported using constructs from the Theoretical Framework of Acceptability (TFA) by Sekhon, Cartwright (22)

| Acceptability construct | HABIT findings in relation to the acceptability framework |
| --- | --- |
| Affective attitude | - The health visitors valued the evidence-based oral health training - Parents felt it was appropriate to discuss their child’s oral health with their health visitor and were accepting of the guidance they provided |
| Burden | - Some parents had prioritised other things to discuss, which may have decreased the amount of time taken to deliver the intervention |
| Ethicality | - Fits with health visitors’ current practice of delivering oral health, but provides a more structured and comprehensive approach - Integrates well into family life |
| Intervention coherence | - Health visitor diaries highlighted that there was variance in intervention duration - Some health visitors did not use the action plan within their visit |
| Opportunity costs | - Health visitors did not think it was appropriate to introduce the action plan to parents who were perceived to be ‘educated' |
| Perceived effectiveness | - Parents felt reassured that they were doing the right thing. - Health visitors felt the training was effective and empowered them to undertake effective oral health conversations |
| Self-efficacy | - The intervention provided confidence for first-time mothers - When the child became- more resistant at a later age, parents felt less confident about toothbrushing |
