# Supplementary material for: HABIT (Health visitors delivering Advice in Britain on Infant Toothbrushing): a qualitative exploration of the acceptability of a complex oral health intervention
Source: BMC Prim Care. 2022 Mar 26;23:55. doi: 10.1186/s12875-022-01659-1 (PMC8962587; doi:10.1186/s12875-022-01659-1)
Supplement: Supplementary file 5 — Additional file 5. A tabulated summary of themes and subthemes. [file 12875_2022_1659_MOESM5_ESM.docx]

Additional file 5: A tabulated summary of themes and subthemes

|  | **Themes** | **Sub-themes** |
| --- | --- | --- |
| Parents | 1. Health visitor as a “trusted” person | - 1. HV was the right person to deliver the intervention   2. Preference for a consistent HV to enable rapport and insight   3. Feeling comfortable with HV |
|  | 1. Importance of reassurance | - 1. Reassurance for “doing the right thing” even when optimal behaviours already established   2. Reassurance especially important for a first-time mum |
| Health visitors | 1. The usefulness of training/resources | - 1. A consistent approach   2. Usefulness of resources   3. Areas for improvement |
|  | 1. Challenges changing complex family norms | - 1. Toothbrushing is not always a priority   2. Behaviour is ingrained into the culture |
| Parents and Health visitors | 1. Timing of the intervention is important | - 1. Anticipatory guidance is needed   2. Habits have already been established   3. What is the Minimum I Need to Do? |
|  | 1. Integration of intervention | - 1. Integration into family life/practice   2. Passing on information to other family members   3. Integrating into the wider community/environment or context |
